# Gluteal Compartment Syndrome Secondary to Pelvic Trauma

**DOI:** 10.1155/2016/2780295

**Published:** 2016-08-08

**Authors:** Fernando Diaz Dilernia, Ezequiel E. Zaidenberg, Sebastian Gamsie, Danilo E. R. Taype Zamboni, Guido S. Carabelli, Jorge D. Barla, Carlos F. Sancineto

**Affiliations:** Institute of Orthopaedics “Carlos E. Ottolenghi” Italian Hospital of Buenos Aires, C1199ACK Buenos Aires, Argentina

## Abstract

Gluteal compartment syndrome (GCS) is extremely rare when compared to compartment syndrome in other anatomical regions, such as the forearm or the lower leg. It usually occurs in drug users following prolonged immobilization due to loss of consciousness. Another possible cause is trauma, which is rare and has only few reports in the literature. Physical examination may show tense and swollen buttocks and severe pain caused by passive range of motion. We present the case of a 70-year-old man who developed GCS after prolonged anterior-posterior pelvis compression. The physical examination revealed swelling, scrotal hematoma, and left ankle extension weakness. An unstable pelvic ring injury was diagnosed and the patient was taken to surgery. Measurement of the intracompartmental pressure was measured in the operating room, thereby confirming the diagnosis. Emergent fasciotomy was performed to decompress the three affected compartments. Trauma surgeons must be aware of the possibility of gluteal compartment syndrome in patients who have an acute pelvic trauma with buttock swelling and excessive pain of the gluteal region. Any delay in diagnosis or treatment can be devastating, causing permanent disability, irreversible loss of gluteal muscles, sciatic nerve palsy, kidney failure, or even death.

## 1. Introduction

Gluteal compartment syndrome (GCS) is extremely rare when compared to other anatomical regions, such as the forearm or the lower leg [1]. Several nontraumatic causes have been described. According to the literature, most cases present on patients with a history of drug abuse (alcohol or opioid intoxication) causing prolonged immobilization due to loss of consciousness [2–5]. Other causes such as anticoagulation, obesity, and incorrect position during orthopedic or urological surgeries with long operative time and epidural anesthesia have also been reported. However, GCS secondary to pelvic trauma has rarely been reported in the literature [6–8].

Clinical findings are similar to those of other compartment syndromes such as excessive pain (usually out of proportion to the injury), paresthesia, and tense compartments. Other possible findings range from sciatic nerve palsy to massive rhabdomyolysis (RM), acute kidney failure, multiple organ dysfunction syndrome, and even death. Most authors suggest an intracompartmental pressure threshold of 30 mmHg as the threshold for initiating treatment, but clinical diagnosis remains the best way for evaluating the patient. The measurement of gluteal compartment pressure may be especially helpful in unresponsive patients where symptoms like pain or paresthesias cannot be assessed. Image studies, such as MRI, CT scan, and ultrasound, are often omitted in order to avoid delays in treatment. The gold standard for treatment is emergent fasciotomy [9–11].

We present a case of gluteal compartment syndrome secondary to an anterior-posterior compression pelvic ring injury with a left sacroiliac dislocation and pubic symphysis diastasis without fracture.

## 2. Case Report

A 70-year-old Caucasian man with no prior medical history suffered a pelvic trauma after being run over by a truck, sustaining an anterior-posterior compression pelvic injury. Primary stabilization with pelvic external fixation and damage control was performed in another institution. Twelve hours after the accident, the patient was admitted to our emergency department.

**Figure 1:**
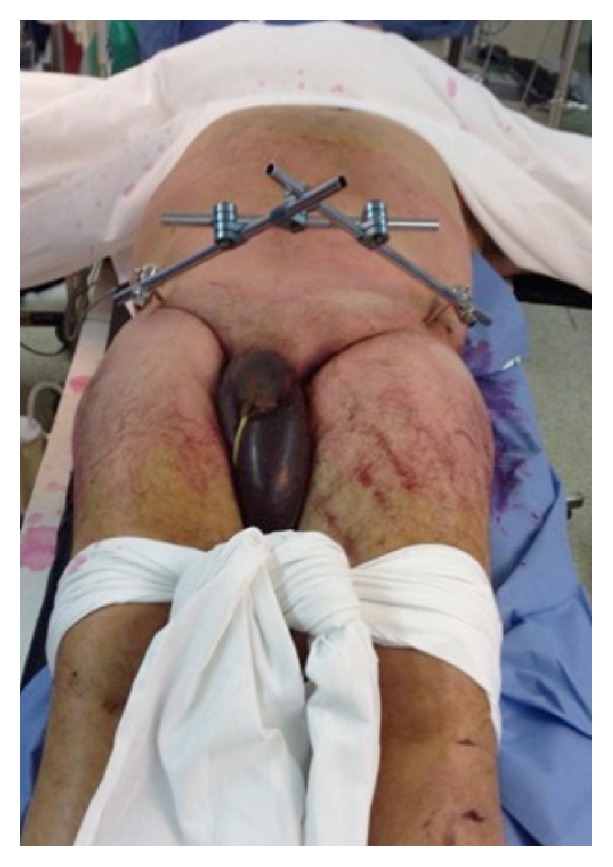
Clinical photograph showing skin marks of truck wheels on the left thigh, extensive scrotal hematoma, and swelling.

**Figure 2:**
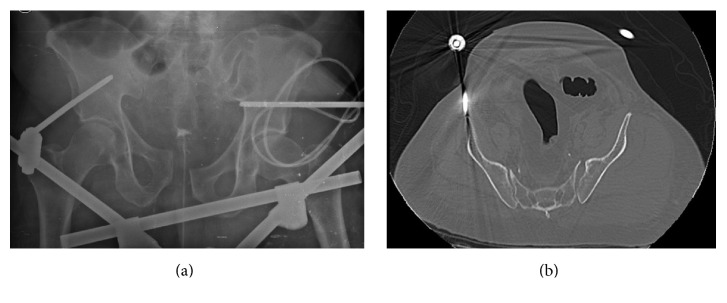
(a) Anteroposterior radiograph of the pelvis showing pubic symphysis diastasis. (b) CT scan axial view confirms pelvic ring injury due to sacroiliac joint subluxation without bone involvement.

The patient was hemodynamically stable and responsive, complaining of pain on the lateral and posterior regions of the left buttock, accompanied by left ankle extension weakness. Physical examination revealed a truck wheel-shaped bruise on the left thigh, scrotal hematoma, and swelling (Figure 1). Left ankle extension showed active movement against gravity, with some weakness against resistance. Arterial pulses were intact, but sensory and motor deficits were consistent with left sciatic nerve palsy. Pelvic radiographs and computed tomography showed a traumatic disruption of the pelvic girdle without bony injury. Left sacroiliac dislocation and pubic symphysis diastasis were still evident despite the external fixation (Figure 2).

Admission laboratory results showed increased levels of creatine phosphokinase (CPK) and lactate dehydrogenase (LDH), suggesting muscle death and tissue damage. He subsequently lost left ankle flexion and extension, suggesting sciatic nerve palsy. One hour after his arrival and considering the clinical findings, diagnosis of GCS was made and urgent fasciotomy was indicated.

**Figure 3:**
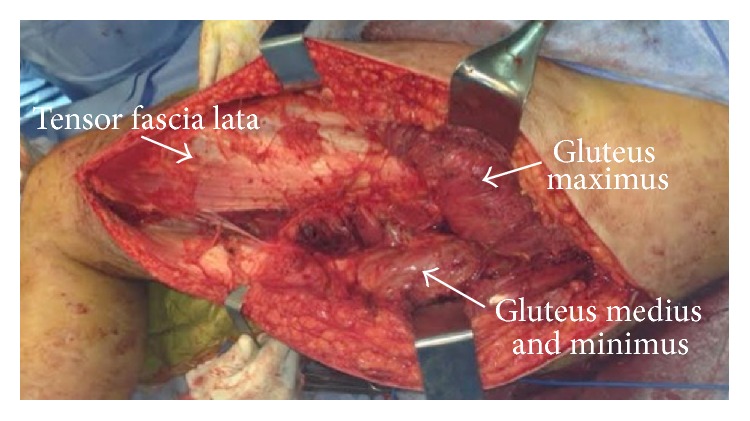
Intraoperative image showing the urgent fasciotomy and the decompression of the three gluteal compartments (tensor fascia lata, gluteus minimus and medius, and the gluteus maximus) with distal extension through the left tight.

Prior to the surgery, an intracompartmental pressure of 46 mmHg was measured in the gluteus maximus compartment, confirming the diagnosis of gluteal compartment syndrome. Urgent fasciotomy was performed to decompress the three muscle compartments of the gluteal region (gluteus maximus, gluteus medius/minimus, and the fascia lata) (Figure 3). Vaccum assisted closure was applied for wound management.

Before surgery, the patient developed acute kidney failure with anuria along with increased serum values of urea and creatinine, requiring hemodialysis after the procedure. Once the patient was stable, open reduction and internal fixation by anterior and posterior approaches were performed. Laboratory markers (urea, CPK, Cr, and LDH) returned to normal values within the following month, after which the patient was discharged. Three months after initial trauma, the patient recovered normal kidney function but continued with sciatic nerve palsy. At one-year follow-up, the patient persists with neurological deficit according to the Medical Research Council (MRC). He had 1/5 strength with testing of the anterior tibialis, musculus peroneus longus, and musculus peroneus brevis.

## 3. Discussion

Compartment syndrome (CS) is a surgical emergency caused by a microvascular phenomenon due to increase of the interstitial pressure in a nonexpandable musculoskeletal compartment. It results in soft tissue ischemia causing cellular hypoxia and death [9, 12]. Whitesides et al. reported that four-hour ischemia leads to irreversible muscle damage [2, 13]. Neurons are even more sensitive to hypoxia, and compromise of nervous tissue may occur in just 33 min [1, 13]. Other authors have reported that eight hours of muscle ischemia causes irreversible damage [13]. For this reason, early compartment syndrome identification remains the cornerstone, as a delay in the diagnosis can be disastrous for the patient and can lead to severe metabolic and neurological complications.

The incidence of compartment syndrome (CS) in the upper and lower limbs has been well documented but has rarely been reported in the literature as occurring in the gluteal region [1]. There are three compartments in this region, which in order of appearance (from lateral to medial) are as follows: tensor fasciae latae, gluteus medius and minimus, and finally gluteus maximus [1, 2, 6, 14]. The release of these three compartments is vital in the treatment of CS in the gluteal region [9].

The most common causes of GCS are related to prolonged local pressure on the gluteal muscles, usually from improper positioning during long surgical procedures or unconsciousness due to alcohol or drug abuse [2–5]. Obesity, unconsciousness, and epidural anesthesia are associated risk factors and can obscure the diagnosis. There are also some reports of GCS associated with the use of statins [15, 16], as a complication of hip surgery [17], intramuscular injections [18], Ehlers-Danlos syndrome [19], infection [20], superior gluteal artery rupture, sickle cell disease, and trauma, with the latter rarely being associated with this pathology [6–8]. Henson et al. performed a systematic review and found seven articles with a total of 28 cases [21]. Causes included prolonged immobilization (50%), post-total joint arthroplasty with epidural anesthesia (21%), trauma (21%), and necrotizing fasciitis (7%) [21].

Measurement of compartment pressures (CP) may be helpful, especially in unconscious patients. Normal intracompartmental pressure is 0–8 mmHg in adults. Pain and paresthesias appear with a pressure above 20–30 mmHg [22]. Pressures greater than 30 mmHg are suggestive of CS and fasciotomy is indicated [14]. Despite this, there is no consensus in the literature about the threshold that is an indication for surgery. If there is clinical suspicion, surgical intervention must be performed immediately [14]. Early treatment with fasciotomy considerably improves the chances for full recovery [10, 11].

In late stages of the GCS, ischemic changes occur in the sciatic nerve [23]. Symptoms progress through paresthesias, paresis, anesthesia, and, finally, palsy with loss of peripheral pulses [24]. In the pelvis, the sciatic nerve runs between the gluteus maximus and the pelvis external rotator complex, making it susceptible to compression by swelling of the gluteal muscles. This may result in a compression-induced neuropathy [25]. Hargens et al. showed that the time required to produce peripheral nerve compromise is inversely proportional to intracompartmental pressure [26]. More than half of the patients suffer neurological symptoms due to sciatic nerve damage and symptoms persist if the treatment is delayed [3]. This hypothesis could explain the sciatic nerve palsy sequelae that our patient suffered, which could have probably been related to delayed diagnosis and treatment.

Another possible complication of the GCS is crush syndrome, also known as traumatic rhabdomyolysis or Bywater's syndrome. Crush syndrome is the systemic consequence of severe rhabdomyolysis characterized by significantly elevated values of creatinine and urea, with myoglobin present in the urine and hyperkalemia. The necrotic muscle causes cellular death with release of myoglobin and potassium to the extracellular space and blood stream. The resulting hyperkalemia causes acidosis, and myoglobin deposits in the distal renal tubules, which may result in acute kidney failure [9, 27–29]. The treatment for crush syndrome must be aggressive in order to prevent further kidney damage, and the treatment includes fluid resuscitation and urine alkalinization [10].

GCS is an extremely rare condition that can be easily overlooked, especially in obese or unconscious patients. Due to patient responsiveness in the present case, the swelling and tautness were easily recognized and diagnosed immediately, allowing a prompt treatment. However, it is possible that the 12-hour delay in the patient's arrival to our institution may have been the cause of complications such as kidney failure and sciatic nerve palsy.

Trauma surgeons must be aware of the possibility of GCS in patients who have an acute pelvic trauma with swelling and excessive pain of the gluteal region. It has a high morbidity rate and must be kept in mind in the differential diagnosis for patients with pelvic trauma. Any delay in diagnosis or treatment can be devastating, causing permanent disability, irreversible loss of gluteal muscles, sciatic nerve palsy, or even end-stage kidney failure. This case highlights the importance of early diagnosis and treatment of this uncommon condition.
